# Developing a task-sharing psychological intervention to treat mild to moderate symptoms of perinatal depression and anxiety in South Africa: a mixed-method formative study

**DOI:** 10.1186/s13033-021-00443-5

**Published:** 2021-03-15

**Authors:** Sonet Boisits, Zulfa Abrahams, Marguerite Schneider, Simone Honikman, Debra Kaminer, Crick Lund

**Affiliations:** 1 Alan J Flisher Centre for Public Mental Health, Department of Psychiatry and Mental Health, University of Cape Town, 46 Sawkins Road, Rondebosch, Cape Town, 7700 South Africa; 2 Perinatal Mental Health Project, Alan J Flisher Centre for Public Mental Health, Department of Psychiatry and Mental Health, University of Cape Town, 46 Sawkins Road, Rondebosch, Cape Town, 7700 South Africa; 3 Department of Psychology, University of Cape Town, Rondebosch, Cape Town, 7701 South Africa; 4 King’s Global Health Institute, Centre for Global Mental Health, Health Service and Population Research Department, Institute of Psychiatry, Psychology and Neuroscience, King’s College London, De Crisping Park, London, SE5 8AF UK

**Keywords:** Perinatal, Depression, Anxiety, Task-sharing, Psychological intervention, Primary health care, Low- and middle-income countries

## Abstract

**Background:**

Symptoms of depression and anxiety are highly prevalent amongst perinatal women in low-resource settings of South Africa, but there is no access to standardised counselling support for these conditions in public health facilities. The aim of this study is to develop a task-sharing psychological counselling intervention for routine treatment of mild to moderate symptoms of perinatal depression and anxiety in South Africa, as part of the Health Systems Strengthening in sub-Saharan Africa (ASSET) study.

**Methods:**

We conducted a review of manuals from seven counselling interventions for depression and anxiety in low- and middle-income countries and two local health system training programmes to gather information on delivery format and common counselling components used across task-sharing interventions. Semi-structured interviews were conducted with 20 health workers and 37 pregnant women from four Midwife Obstetric Units in Cape Town to explore perceptions and needs relating to mental health. Stakeholder engagements further informed the intervention design and appropriate service provider. A four-day pilot training with community-based health workers refined the counselling content and training material.

**Results:**

The manual review identified problem-solving, psychoeducation, basic counselling skills and behavioural activation as common counselling components across interventions using a variety of delivery formats. The interviews found that participants mostly identified symptoms of depression and anxiety in behavioural terms, and lay health workers and pregnant women demonstrated their understanding through a range of local idioms. Perceived causes of symptoms related to interpersonal conflict and challenging social circumstances. Stakeholder engagements identified a three-session counselling model as most feasible for delivery as part of existing health care practices and community health workers in ward-based outreach teams as the best placed delivery agents. Pilot training of a three-session intervention with community-based health workers resulted in minor adaptations of the counselling assessment method.

**Conclusion:**

Input from health workers and pregnant women is a critical component of adapting existing maternal mental health protocols to the context of routine care in South Africa, providing valuable data to align therapeutic content with contextual needs. Multisector stakeholder engagements is vital to align the intervention design to health system requirements and guidelines.

## Background

There is a high prevalence of Common Mental Disorders (CMDs) such as anxiety and depression amongst women in the perinatal period (from pregnancy to 1 year after delivery), in low- and middle-income countries (LMICs) [1–4]. A systematic review and meta-analysis of Perinatal Common Mental Disorders (PCMDs) in LMICs reported 19–25% prevalence of perinatal depression and 26–35% prevalence of symptoms of anxiety, compared to HICs where prevalence ranged between 7–15% and 14–20%, respectively [1, 3]. In South Africa, a community-based cluster randomised controlled trial (RCT) conducted in two peri-urban settlements of Cape Town reported that 39% of pregnant women suffered from symptoms of depression [5], while a cross-sectional study of pregnant women in an urban area of Cape Town reported a 23% prevalence of any diagnosable anxiety disorders [6].

Risk factors such as poverty, an unintended pregnancy, violence, lack of support and a history of mental illness are considered consistent predictors of depression and anxiety, with multiple stressors increasing the risk [2, 6, 7]. Untreated symptoms of depression and anxiety during pregnancy may result in similar symptoms after birth [8] and hold negative consequences for infant development and mother–child bonding [9–11]. Even so, evidence suggests that perinatal depression is still under-detected and undertreated in LMICs [3] and standardised psychosocial treatment at primary healthcare level is lacking [12]. Therefore, developing an effective, scalable, and culturally sensitive intervention to treat perinatal depression and anxiety, particularly for diverse, low-resource communities in LMICs, is a public mental health priority.

To promote access to mental health care in LMICs, it is recommended that treatment be integrated into routine primary health care practices [13]. The overwhelming shortage of human resources to deliver mental health care, along with a need for cost-effective and scalable mental health interventions has encouraged task-sharing (when a non-specialist health worker is trained to deliver a service under the guidance of a specialist) as an alternative workforce strategy [14–17]. Over the last decade, various maternal mental health studies in South Africa have identified a need to develop and integrate standard detection, referral and treatment practices into routine primary care, including through task-sharing interventions [5, 18–21]. In turn, community health workers have been identified as a suitable cadre to deliver culturally appropriate psychosocial support, on condition of well-developed training programmes and regular supervision [22, 23].

These research innovations have been linked with recent policy and service developments. Consistent with the adoption of the National Mental Health Policy Framework and Strategic Plan 2013–2020, designed to improve the quality and accessibility of mental health care in South Africa, a standardised maternal mental health screening protocol was introduced at primary health care level [24–27]. In line with the re-engineering of South Africa’s primary health care initiative, a Ward-based Primary Health Care Outreach Team (WBOT) strategy was introduced. This is the first national community health worker (CHW) programme, designed to extend essential health services from the facility to the community [28]. WBOTs, comprising of professional and lay health workers, are linked to contracted not-for-profit organisations (NPOs) and provide support, aimed at the prevention and management of TB/HIV, hypertension, diabetes and maternal and child health, in patients’ homes [29].

Despite the progress made with a facility-based maternal mental health screening protocol and maternal and child health support (which consists of supporting perinatal women with general, primary-level child health and development-related matters) at household level, access to routine mental health counselling for perinatal women who screen positive for mild to moderate symptoms of depression and anxiety is still lacking in South Africa [30]. This often leads to treatment delays or inconsistent maternal mental health support. In line with strengthening existing health system processes, task-sharing mental health counselling support can ideally follow a four-phase approach: facility-based screening by a professional nurse, referral to community-based services, delivery of a short-term evidence-based intervention at household level and referral back to the facility if further specialist support is required.

This paper draws on the formative diagnostic phase of the Health Systems Strengthening in sub-Saharan Africa (ASSET) study, a multi-country study with an overarching goal to strengthen health care systems in Sub-Saharan Africa (healthasset.org). In South Africa, one of the study aims is to promote public maternal mental health care through strengthening screening, referral and treatment (including counselling) services, in collaboration with the Western Cape Department of Health (WCDoH). This study aims to describe the processes involved in developing an evidence-based, task-sharing psychological counselling intervention supported by standardised training and supervision guidelines [31], integrated into current health systems and adapted to local settings, to strengthen routine maternal mental health care.

## Method

### Setting

We conducted the study in four Midwife Obstetric Units (MOUs) situated in Community Health Centres (CHCs) in Cape Town. These facilities serve four low socio-economic communities situated in urban and peri-urban areas in the Cape Town Metropolitan health district, located between 20 and 40 kms from the city centre, with a population size ranging between approximately 33,200 to 44,400 per facility catchment area [32, 33]. The majority of the individuals in these communities describe themselves as ‘coloured’. In South Africa the term ‘coloured’ is commonly used to refer to individuals who are of mixed-race ancestry [34]. While the predominant language is Afrikaans, isiXhosa- and English-speaking community members from neighbouring areas also attend these facilities. Antenatal care is predominantly provided at MOUs by nursing staff and non-specialist health workers. The Department of Health WBOTs, managed by professional nurses, receive referrals from designated CHCs and provide community-based integrated health care services at household level.

### Study design

**Fig. 1:**
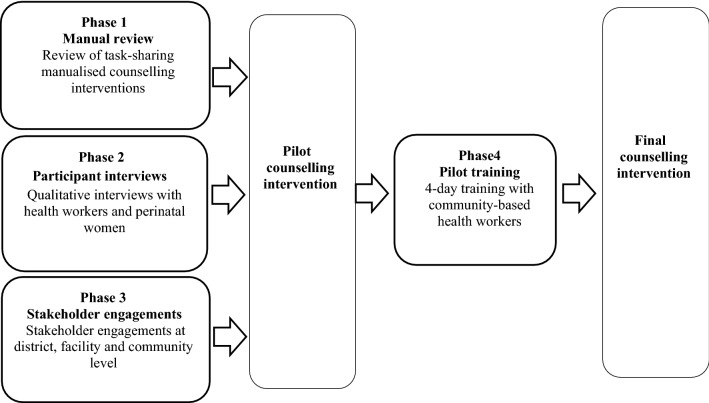
Overview of the four-phase study process

We used a qualitative study design with triangulation of data from multiple sources to inform the development of a task-sharing counselling intervention (see Fig. 1).

We used a four-phased process. The first phase involved a review of task-sharing counselling intervention and health system training manuals. For the second phase we conducted qualitative interviews with health workers and pregnant women; and the third phase entailed engagements with the Western Cape Department of Health (WCDoH) managers from each of the four health sub-districts, facility-based management teams and NPO co-ordinators. Findings from phase one to three informed the draft version of the counselling intervention and training manual. Phase four then consisted of a four-day classroom-style pilot training with CHWs and supervisors from one NPO, followed by a feedback session. Learnings from the pilot study informed the final version of the intervention design and training manual.

### Participants

For phase two, we recruited pregnant women and health workers through purposive sampling at four MOUs. At each facility, pregnant women were screened by trained fieldworkers. Those women who screened positive for symptoms of depression and/or anxiety (using the Edinburgh Postnatal Depression Scale) [35] or experiences of violence (using a bespoke questionnaire), during the recruitment period, were invited to participate in a 30-min audio-recorded semi-structured interview. The mean age of the pregnant women was 29 years. Half of them were either employed or self-employed while the other half were unemployed and dependent on family to meet their basic needs. All except one woman had experienced a previous pregnancy.

Professional and lay facility-based health workers who provided care to pregnant women, were invited to participate in individual 30 to 60-min audio recorded interviews. Twenty health workers [including operational managers, antenatal care nurses, lay counsellors (consisting of HIV and breastfeeding counsellors), health promotion officers and mental health nurses] participated in the study.

In phase four we selected CHWs and supervisors from one NPO (providing community-based support to the pilot study site) to take part in training for the intervention protocol. The inclusion criteria for CHWs were: minimum of three years’ experience, completion of the WBOT in-service training programme and interest in mental health counselling. Five CHWs with two of their supervisors (professional nurses) met the inclusion criteria and we invited them to a 3-day class-room style training. The supervisors were then invited to participate in an additional day of supervision training.

### Data collection

In phase one we reviewed two systematic reviews of task-sharing psychological treatments for low- and middle-income countries. In addition, we reviewed and reported on the content of seven task-sharing counselling manuals that met our inclusion criteria. The inclusion criteria for the manual review were: individual perinatal psychological interventions for the treatment of depression and anxiety, designed for delivery by non-specialist health workers, in LMICs with proven effectiveness. First, we inspected studies included in the Clarke et al. systematic review of perinatal psychosocial task-sharing interventions for CMDs [15]. Second, we inspected studies in the Singla et al. systematic review of task-sharing psychological interventions for LMICs [15, 36]. In addition, we supplemented our findings from these reviews with maternal mental health intervention manuals developed for the South African context, obtained from experts in the field such as the ASSET principal investigator and co-investigators of the African Focus on Intervention Research for Mental Health (AFFIRM) and Perinatal Mental Health Project (PMHP)). Further to expert recommendations, we sought to broaden the scope of the manual review to include broader task-sharing depression counselling treatments and interventions that integrated transdiagnostic evidence-based task-sharing approaches adapted to local cultures and contexts in LMICs. District and facility-based health system stakeholders shared in-service training programme guides, that allowed us to explore standard training methodologies and guidelines. The final manual review included six manualised evidence-based task-sharing counselling interventions for LMICs (AFFIRM, Thinking Health Programme, Friendship Bench, Healthy Activity Programme, Problem Management Plus, Trauma-Focused Cognitive Behavioural Therapy) [37–42] and one basic perinatal counselling skills guide for health workers in South Africa (PMHP basic counselling guide for health workers) [19]. In addition, two health systems in-service training guides for HIV and community-based counselling were included in the review.

The conceptual model for understanding the cultural and contextual determinants and manifestations of depression and anxiety was built on two areas of previous work: (1) on Kleinman’s theory of explanatory models that suggests that social and cultural contexts have a strong influence on the explanatory models that individuals or groups use to make sense of their illness experience [43, 44] and (2) on an understanding of the critical role of the social determinants of mental health in shaping the mental health of populations [45]. Therefore, it is essential to consider context-relevant social determinants and the mental health views of health service providers and users when planning a mental health counselling intervention.

In phase two, we conducted semi-structured interviews with 37 pregnant women and 20 health workers at the four MOU sites from September 2018 to February 2019. The interviews were conducted in a private room in the participants’ preferred language (English, Afrikaans or isiXhosa). Interview questions for pregnant women included: the participant’s understanding and experiences of depression and anxiety, perceived causes of distress, experiences of violence or abuse, perceived support and personal coping methods. Interview questions for health workers included: the participant’s work experience and mental health and counselling training, understanding and description of depression and anxiety, perceived causes of these symptoms and common forms of violence as observed in their work with women during and after pregnancy (perinatal period). In phase three we engaged various stakeholders through presentations, workshops and meetings. The stakeholders included: (1) a DoH working group that consisted of managers of each of the four health districts in the city of Cape Town, (2) the facility- and community-based managers, and (3) managers and staff from the four MOUs. Feedback from these interactions were recorded in meeting minutes and notes. In phase four, we conducted a four-day pilot training that consisted of 3 days counselling training with CHWs and one-day training with supervisors. The training programme included activities such as scenario exercises, trainer demonstrations, participant role-plays and group discussions. Through these activities, the trainer was able to observe how well the participants engaged with the manual content, the terminology used and the level of difficulty of counselling concepts. Two additional post-training feedback meetings with supervisors informed further content adjustments to the intervention protocol.

### Data analysis

For each of the manuals identified in the manual review, we extracted data using the following preselected categories: therapeutic goals, counselling techniques, number of sessions, frequency of delivery (monthly or weekly), delivery method (group or individual) and additional in-session techniques. For phase two, we used a thematic framework analysis approach to analyse the interview transcripts [46]. The semi-structured interviews with health workers and pregnant women were transcribed verbatim by experienced bilingual transcribers. Interviews conducted in Afrikaans and isiXhosa were first translated into English before analysis began. The thematic framework was guided by the interview topic questions and further emergent themes were captured through in-depth reading of the transcripts. Transcripts and data were managed using NVivo 12 Pro qualitative data analysis software (QSR International Pty Ltd). Three researchers were involved in the coding process of the health worker interviews and each analysed a third of the transcripts. Two researchers were involved in the coding process of pregnant women interviews and each analysed half of the total number of transcripts. In both coding processes, 10% of all transcripts were randomly selected for double-coding and analysed to establish inter-coder reliability. In phase three, we incorporated feedback from managers and healthcare workers into the draft version of the counselling intervention. This version was then used during the pilot training. In phase four, we documented the pilot training findings in the form of training observations by the trainer during counselling role-plays, verbal participant feedback during group discussions, a debriefing session with supervisors after training and personal training notes. Minor manual content adjustments were made throughout the training process and tested during training. Learnings from the pilot study informed the development of the final version of the counselling intervention.

## Results

### Phase 1: Manual review

The data extraction from the manual review is summarised in Table 1. The duration of the evidence-based treatment programmes ranged between 6 to 16 structured counselling sessions, while the basic counselling skills guide included a total of 2 to 3 unstructured counselling sessions. The length of a session, where reported, ranged from 30 to 90 min. In all the interventions, the delivery method was individual sessions and the frequency of delivery varied between weekly and monthly sessions. A problem-solving approach (or adaptations thereof commonly referred to as ‘problem management’), psychoeducation and basic counselling skills (both verbal and non-verbal), were integrated into all the interventions in the review, while Behavioural Activation was present in four. These components were therefore identified as preferred elements for inclusion in our draft counselling intervention design. We further incorporated popular adjunct components that included praising openness and validating feelings, relaxation techniques, engaging social support and enhancing safety. We found that health system in-service training methodologies were based on adult learning principles and integrated various scenario-based training activities. Basic counselling modules formed part of health system training programmes and mainly focused on verbal and non-verbal communication skills and basic problem-solving to address health and psychosocial challenges.

**Table 1:** Characteristics of task- sharing psychological interventions

Intervention description	Treatment focus	Evidence-based treatment approach	Total sessions	Length of a session	Frequency of sessions	Delivery method and setting	Additional components
Evidence-based task sharing counselling interventions							
AFFIRM (Lund, South Africa, 2019) To equip CHWs with skills to offer counselling support to mothers in low resources settings	Perinatal Depression SA	Problem-solving Behaviour activation Cognitive behaviour therapy	6	60 m	bi-monthly	Individual home-based visits	Psychoeducation Relaxation Basic counselling skills Assessment guideline Safety guideline
THINKING HEALTHY (Rahman, Pakistan, 2008) To equip lay health care workers with skills to assist to take better self-care, bond with their baby and enhance social support	Perinatal Depression LMICs	Cognitive behaviour therapy Problem-solving	16	45–60 m	Varied—Integrated into routine visit	Individual, home-based visits	Psychoeducation Relaxation Family engagement Mobilising social support Basic counselling skills
HEALTHY ACTIVITY (Patel, India, 2017) To strengthen counselling skills and better equip lay health workers to deliver a culturally appropriate treatment	Depression	Behaviour activation Problem-solving	6–8	30–40 m	Spread over 2–3 months	Individual, facility -based visits	Psychoeducation Basic counselling skills and collaborating with the client Safety assessment Involvement significant others (SO)
THE FRIENDSHIP BENCH (Chibanda, Zimbabwe, 2017) To provide lay health workers with the skills to deliver a problem-solving therapy intervention for common mental disorders related to HIV	Depression and anxiety	Problem-solving	6	60 m	Spread over 2–3 weeks	Individual, facility-based visits	Basic counselling skills
Problem Management Plus (PM +) (WHO, 2016) To provide lay health workers with the skills to deliver a low intensity psychological intervention to improve a client’s ability to manage their own emotional distress	Depression anxiety and stress	Problem-solving Behavioural activation	5	90 m	weekly	Individual, home-based visits	Basic counselling skills Praising openness, validating feelings Social support Relaxation techniques
Trauma Focused CBT (Murray, 2015) To provide lay counsellors with the skills to deliver an evidence-based trauma treatment programme	Depression anxiety and PTSD	Problem-solving Behaviour activation Cognitive behaviour therapy	8	90 m	weekly	Individual, facility or community-based visits	Psychoeducation Praising openness, validating feelings Social support Relaxation techniques Safety
Basic counselling support and containment for distress							
Basic Counselling Skills (PMHP, 2011) To educate and empower maternal care health workers with the skills to support perinatal women in distress	Maternal mental health	Person-centred Problem management	2–3	NR	NR	Individual, facility or community-based visits	Principles of basic counselling Role of the helper Psychoeducation Caring for the counsellor
Department of Health in-service training programmes for Community Health Workers							
HIV Counselling training for lay counsellors	HIV and related symptoms	Problem-solving method integrated into training programme design	NR	NR	NR	Individual, facility based	Psychoeducation Basic counselling skills Pre- and post-counselling
Ward-based Community Health worker Training	Seven core skills	Problem-solving method integrated into training programme design	NR	NR	NR	Individual, community based	Health Promotion Confidentiality Communication skills Screening Tracing Psychosocial support

NR: Not Reported

### Phase 2: Participant interviews

Professional and lay health workers identified and described symptoms of depression and anxiety and their perceived causes of these symptoms amongst pregnant women. Pregnant women described their experiences of depression and anxiety, the causes related to their unique circumstances and personal ways of coping with distress. In addition, they expressed their preferred place for counselling and their perceptions of their community’s view on mental illness. All participant descriptions of symptoms and experiences of depression, anxiety and violence informed the intervention content. Commonly used words and expressions used by both health workers and pregnant mothers were integrated into the overall intervention design and counselling content to promote acceptability.

The findings are discussed further in the following sections, where the proportions of women who report certain themes are presented; the denominator varies at times, depending on how many study participants were asked a specific question.

#### Descriptions of depression and anxiety

Professional and lay health workers commonly (15 of 20) identified signs of perinatal depression in behavioural terms, as illustrated in the following example, *“she is not getting out of the bed, or do not do exercise and sleeps the whole day. So that is for me an indication that this patient needs encouragement”* (Professional health worker MC03). Similarly, the majority of pregnant women (29/37) described symptoms of depression as a change in usual behaviour, for example, *“I am lazy now to do things…I do not feel I can do the washing, the dishes. I am lazy”* (Pregnant woman HHP01). While professional health workers mostly described symptoms of depression in clinical terms (6/9) some used words such as feeling *“down and low”* (3/9) or various descriptions such as *“you can’t face a new day, everything is just too much”* (Professional health worker BL05). Lay health workers (8/11) mostly demonstrated their understanding by using descriptions or metaphors, for example, “*the person is in a dark hole and can’t get out*” (Lay health worker BL03) and *“it is something that crawls slowly on you and you are not aware of it”* (Lay health worker BL01). Pregnant women commonly (9/20) described feeling depressed as being “*sad or unhappy*” while others (6/20) used the word “*stressed*” interchangeably to describe either depression or anxiety, for example, *“I think when you have depression you have stress too much”.* Some pregnant women struggled to describe symptoms (8/29), as one woman described, *“I have asked myself that question [what caused this feeling] a lot of times because as I told you, I wake up like that”* (Pregnant woman MCP08). For symptoms of anxiety, professional health workers associated a variety of physiological responses (8/19) such as “*shortness of breath and palpitations*” (Professional health worker HP05) while lay health workers were less specific about the difference between anxiety and depression. For example, as explained by one lay health worker, *“anxiety is similar to depression; you can see they are uncomfortable and itchy”* (Lay health worker BLO2).

#### Perceived causes of symptoms and coping methods

We wanted to understand pregnant women’s perceptions of what was causing them distress. Results indicated that strong precipitating factors of depression and anxiety were interpersonal conflict (26/37) between themselves and family members or a partner, difficult social circumstances (24/37) such as unemployment and unintended pregnancies, exposure to violence or abuse (24/37) and a general lack of support or judgement from family or a partner (12/37). Most pregnant women experienced more than one stressor. These findings were verified by the perceptions of health workers, as demonstrated in feedback from two professional health workers, *“we often hear, I am unemployed, my husband is unemployed, and the pregnancy is unwanted”* (Professional health worker VN02) and *“this is an impoverished community with lots of social issues and difficult circumstances such as housing problems, crime, gangsterism in the community but also as part of the family” (*Professional health worker BL04*).* Pregnant women that reported experiences of domestic violence or abuse mostly reported physical violence (11/24) and verbal abuse (11/24) by a partner or family member, while sexual abuse was less often reported (2/24). Similarly, health workers (12/20) reported *“verbal abuse is first, abuse with the mouth or your tongue"* (Lay health worker BL02). Some health workers (10/20) reported that they often suspected physical abuse when working with perinatal women, however these women were less likely to disclose experiences of physical violence or take further action, as suggested by the following professional health worker, *“some of the ladies just don’t talk about it”* (Professional health worker BL05). Even though one health worker suggested that perinatal women often struggle to cope with depression and anxiety, *“it’s the social circumstances around them, they can’t cope with it, and they don’t talk, they don’t get help”* (Professional health worker HP01). Our findings suggested that most pregnant women (29/37) demonstrated a capacity to cope with their unique circumstances. For example, in one instance, a pregnant woman reported, *“my mommy calms me down when I am stressed. She would tell me I must count to 10, breath in and out, and she would do it with me"* (Pregnant woman MCP06).

#### Location of counselling and community perceptions of mental illness

We were interested in whether women preferred counselling in their homes or at the clinic. We also wanted to know whether community views of mental illness and counselling would be a potential barrier to community-based care. Findings indicated that most women found facility-based counselling more private than their home environments and therefore preferred this setting (19/27). A few women preferred being seen in their homes (4/27) or were indifferent about the counselling venue (4/27). Some women felt that in their community, mental illness was either “misunderstood” (3/8) or viewed by others as “being mad” (4/8). A few were concerned about the opinion of neighbours if they were to receive counselling at home (4/17), as one woman described, “*the people will wonder what is going on”* (Pregnant women HPP07). However, most women were indifferent to the opinion of their neighbours (13/17) e.g., “*I do not care what people say about me…what you think about me, it is your opinion not my opinion”* (Pregnant women HPP07) and “*they like to gossip, even about the pregnancy…you hear there and here a story, but I do not take note of it*” (Pregnant women VNP32).

### Phase 3: Stakeholder engagements

Multi-sector stakeholder engagements led to the refinement of the counselling intervention design. Through engagements with district and facility level health managers, health system challenges such as the scarcity of human resources, workload of facility-based health workers and supervision structures were discussed. A brief task-sharing intervention consisting of three 30–45 min sessions was considered most feasible for existing health system processes. DoH managers indicated that primary health care systems were moving towards a community-based approach and that this involved phasing out facility-based lay health workers. Therefore, CHWs in WBOTs were identified as suitable delivery agents. The benefits of a community-based delivery were that CHWs already had access to pregnant women through routine home-based visits and supervisors were already positioned in a supervisory role. In addition, both CHWs and supervisors received WBOT training, delivered by the WCDoH Master Trainers, that involved seven core community-based service delivery skills: confidentiality and ethical work, health promotion, communication skills, screening, tracing and psychosocial support (empathic listening, containing and referral). Supervisors also received additional supervision skills training to ensure quality care at household level. In line with systems strengthening, we positioned our study to enhance existing WBOT psychosocial support services by introducing a maternal mental health evidence-based treatment component. Therefore, we included WBOT training as a prerequisite for CHW counselling training. Even though we recommended criteria to help select CHWs suitable for training, this was not possible for supervisors. WBOT Outreach Team Leaders (OTLs), consisting of professional nurses, automatically assumed the role of supervisor. Further engagements with one WCDoH training centre and the WBOT training programme lead led to the refinement of the supervision structure and counselling training methodology to best fit existing health processes.

### Pilot counselling intervention

#### Intervention structure

The intervention consists of three 45-min structured individual counselling sessions, delivered weekly by a CHW at household level.

#### Intervention content

The content of the first counselling session was informed by findings from phase two (qualitative interviews) that suggested participants commonly described symptoms of depression and anxiety in behavioural terms. Therefore, we sought to incorporate a behavioural activation component, termed “get moving”, to encourage movement and improve mood as part of the first session. We also sought to integrate a metaphor used by participants into the counselling content. We selected a description that compared the experience of depression and anxiety to that of being “stuck in a hole, with no way out”. This description led to the development of a “depression and worry pit” illustration. The illustration was incorporated into the first session to facilitate psychoeducation and behavioural activation. The illustration compares the experience of depression and anxiety to that of finding oneself in a “low space”, “feeling stuck”, “alone and isolated” or “stressed” (whichever words are used by participants). The illustration is used to explore perinatal women’s unique thoughts and feelings when in the “depression and worry pit” as well as their perceived causes of symptoms of depression and anxiety. In turn, they are encouraged to “move out of the pit” by engaging in activities that provide a sense of joy and accomplishment. While attempting to address mood, session one also sets the foundation for problem-solving therapy (PST) and promotes a sense of self-efficacy. Sessions two and three were informed by common counselling components identified in the manual review and participant feedback that associated the cause of symptoms of social stressors. A problem-solving approach was therefore most appropriate to address the multiple stressors that pregnant women often face due to challenging social circumstances. We integrated the following steps into our intervention design: identifying the problem, thinking of solutions, listing positive and negative aspects of these solutions, selecting the best solution and creating an action plan to review at the next session.

#### Adjunct treatments

The following adjunct treatments, informed by findings from phase one (manual review) were included to promote resilience: mindfulness and relaxation breathing techniques, helpful thinking techniques adapted from CBT [38, 47] and enhancing social support [18]. We also added a managing conflict component, which was not prominent in the manual review, but appeared to be very relevant to this context where interpersonal conflict was cited a precipitant of depression and anxiety in the interviews. We introduced adjunct treatments as an optional component to the counselling process and grouped them as ‘coping skills’.

### Supervision

Regular supervision is an important component of this intervention. Weekly group supervision sessions help to promote fidelity to the intervention and strengthen counselling skills over time. It is beyond the scope of this study to provide a detailed account of the supervision approach and guidelines, but we include some observations made during stakeholder engagements and the pilot training in subsequent sections.

### Phase 4: Pilot training

#### Training structure

The four-day pilot training programme was delivered by a professionally registered mental health practitioner, certified trainer and experienced supervisor of lay counsellors (SB). The format of the training was aligned with health system in-service adult-based learning principles. Active learning was facilitated through scenario-based group discussions, trainer demonstrations, role-plays, and interactive participant feedback sessions.

#### Counselling manual and associated resources

A training manual was developed to support the training of CHWs. Findings from qualitative interviews (phase two) identified additional training needs of CHWs such as basic counselling skills to enhance verbal and non-verbal communication, psychoeducation to promote knowledge of maternal mental health and domestic violence and symptom checks and safety steps to identify at risk cases. The layout of the training manual was structured in a sequence to focus on the above-noted training topics first before introducing the three-session counselling intervention. In the training manual, each counselling session was presented according to the following five-step structure: (1) introduction, that includes building rapport, a symptom check, a safety check; (2) what and why of the session; (3) session goal one; (4) session goal two; and (5) wrapping up the session. Context relevant scenarios and activities were integrated throughout the manual content to demonstrate key counselling concepts. In addition, a quick reference guide for CHWs, consisting of keywords and illustrations, was developed to provide guidance in a session. To standardise the 1-day supervision training, we developed a training guide, informed by existing health systems, in-service supervision structures. As the WBOT supervision structure mainly serves a task-driven function (to check fidelity and maintain quality care), we incorporated educational (ongoing skills development) and supportive (to promote CHW motivation and manage burnout) functions as part of the counselling supervision structure.

#### Participant feedback and observations

Trainer observations during the pilot training identified that participants engaged well with manualised activities and key discussion points. Participants’ feedback suggested that they found the training manual layout user friendly and the content relatable. Participants found the concrete example of a depression and worry pit and coping skills useful, and some related it to their own life experiences or shared the skills that they had learnt during training with family members. All participants were unfamiliar with a structured approach to counselling and mainly associated counselling with empathy and listening skills. Most found a stepwise approach to counselling helpful to reach session goals but felt that they needed more time, training and supervision to practice the application of skills and to boost their confidence. CHWs suggested that the brief nature of the intervention made it possible for them to integrate the three-session model into their existing workload. Most felt that the additional mental health knowledge and counselling skills would improve their overall job performance.

Role-play observations highlighted the following two challenges: (1) CHWs consistently veered ‘off track’ during the ‘information gathering’ (assessment) phase, at the start of each session and (2) CHWs found it difficult to contain a distressed client without giving advice or did the problem solving on behalf of the client instead of allowing the client to lead. These challenges were addressed during training through trainer demonstrations. Even though supervisors were not the delivery agents, they found it helpful to attend the CHW counselling training to engage with the counselling content and enhance their ability to check fidelity during supervision. Due to high workloads, supervisors were unable to engage in one-on-one supervision but felt comfortable to host weekly group supervision sessions. Participant feedback in phase four indicated that they found it helpful to think of supervision as “how can I help you deliver the counselling effectively” versus “how can I help you”. For the latter, standard in-service referral protocols for personal support were discussed.

### Final counselling intervention

Lessons from the pilot study informed the final version of the counselling intervention design and training manual. The idea of a pocket-size reference guide was viewed as a valuable tool to serve as an in-session ‘signpost’ to promote fidelity to key session goals and help CHWs develop skills over time (Table 2). To guide the in-session delivery of the assessment process, the option of a pictorial rating scale to track symptom changes between sessions, instead of asking open-ended questions, was discussed with supervisors. A mood assessment rating scale illustration was designed and incorporated into the existing CHW reference guide. Table 2 provides a breakdown of the final version of the three-session counselling intervention and associated in-session reference guide, designed to support a CHW with the successful delivery of the intervention.

**Table 2:** Overview of final 3-session counselling intervention

Session goals	Approach	In-session techniques	In-session reference guide
Session 1 Developing the counselling relationship, educating and identifying unique symptoms, engaging in activity to improve mood	Behavioural activation “get moving”	Psychoeducation Basic counselling skills Engagement Normalise, validate Engaging coping skill	*Basic assessment* Pictorial rating scale *Common symptoms* List of symptom words *Safety* 3 standard questions Steps to enhance safety *Psychoeducation* Depression and worry pit illustration *Activity scheduling options* Context relevant examples
Session 2 Identifying problems causing distress and finding ways to manage or cope with these problems	Problem-solving (introduction) Identify the problem Think of solutions Positives and negatives Select the best solution Create an action plan Review (next visit)	Basic counselling skills Problem-solving steps Encourage action steps Explore barriers Engaging coping skill	*Basic assessment* Pictorial rating scale *Safety* 3 standard questions Steps to enhance safety *Problem-solving steps* Question guide *Resource guide (referrals)* Local resources
Session 3 Reviewing the action plan and practicing problem-solving skills	Problem-solving (review)	Basic counselling skills Problem-solving steps (review) Encourage action steps Promote daily coping skills and remind of benefits	*Basic assessment* Pictorial rating scale *Safety:* 3 standard questions Steps to enhance safety *Problem-solving steps* Question guide *Resource guide (referrals)*Local resources
Enhancing coping skills (optional)	Mindfulness and belly breathing Helpful thinking Social support Conflict management		

After delivery of the 3 structured sessions, ongoing practice can be reinforced during routine visits. In the case where symptoms do not improve or a safety risk present, it should be discussed with the supervisor for a further referral

## Discussion

This paper describes the development of a perinatal task-sharing, evidence-based psychological counselling intervention that was designed to strengthen existing primary health systems in Cape Town, South Africa. Based on multi-sectoral stakeholder engagements, we found a three-session, home-based intervention with a 45-min session duration, delivered weekly by CHWs, best suited to health system requirements. Informed by the manual review and interviews with pregnant women and health workers, the counselling intervention incorporated a problem-solving approach with components of psychoeducation and behavioural activation, adapted to local settings. Optional adjunct treatments, presented as “coping skills”, were incorporated into the overall counselling design to promote resilience.

To develop a scalable, sustainable, suitable and appropriate intervention we had to consider various factors. First, multi-sector stakeholder engagements were critical to develop an intervention that aligns with health system requirements while addressing low-resourced community needs. Second, we had to consider an intervention flexible enough to handle comorbidity and a range of contextual problems in one unified intervention [42, 48]. Third, the evidence-based approach had to be appropriate to the context and straightforward enough to train CHWs [49, 50]. Fourth, standardised counselling training and supervision had to be integrated into existing health system service processes [31, 51, 52], in a system with limited service capacity [52, 53].

Qualitative work with community stakeholders, that explored the perceptions of mental health and perceived causes of distress, provided rich information and allowed us to align evidence-based treatment components to address real-world challenges and adapt counselling and training content to resonate with the local communities through the use of local expressions [43, 54]. Our findings suggest that social stressors were the major contributors to perinatal depression and anxiety, consistent with other maternal mental health studies in South Africa [5, 6, 37]. This emphasised the need to address life difficulties as part of a psychological intervention [55] as was done in Zimbabwe in a problem-solving task-sharing intervention study that successfully integrated and treated CMDs, associated with HIV, as part of primary health care [56]. Narratives from perinatal women with symptoms of depression indicated that symptoms were commonly experienced in behavioural terms such as a loss of interest in usual activities or lack of energy. Therefore, we found it suitable to integrate a behavioural activation component—a proven effective approach for task-sharing interventions and the treatment of depression [40]. Whereas a Cape Town perinatal evidence-based intervention study’s attempt to incorporate a multifaceted treatment approach (psychoeducation, CBT, BA and PS) to treat depression and address life challenges, had proven too complex and in need of robust training and supervision [37, 48], our intervention was guided by one core therapeutic approach, with standardised training guidelines and scheduled supervision. Informed by findings from phase one to three of our study, problem solving was selected as the core component [39], with aspects of psychoeducation [57] and behavioural activation [40, 58] being integrated into the 3-session counselling intervention. Given the short nature of the intervention design and the fact that newly acquired skills require reinforcement, we recommended that recap sessions be integrated into routine home-based visits to extend the counselling process beyond the three-sessions, albeit in a less structured manner.

Feedback from our pilot training suggested that CHWs required more time to reinforce skills. Supervision is therefore central to our intervention to promote fidelity and help CHWs develop skills and confidence over time [31, 59, 60]. While we are aware that structured supervision programmes remain an ongoing challenge, particularly for upscaling [61], we experienced positive team cohesion between CHWs and WBOT supervisors during our training which suggests that they may be well placed as counselling supervisors.

The need for a national governmental regulatory framework that drives non-specialist health service delivery in South Africa [62] has been addressed through the introduction of WBOTs. This community health worker programme—introduced in the Western Cape in 2019—draws on prescribed guidelines for training, health service delivery and supervision practices that can be taken to scale. This strategy offers a favourable framework to augment existing maternal mental health care that forms part of community-based, health system processes. Through our counselling intervention that includes structured training and supervision guidelines we hope to empower CHWs and supervisors, already in contact with perinatal women at household level, with additional personal and professional skills that may benefit WBOT functions as a whole [49].

This mixed-method study extended maternal mental health services from the facility to the community by collaborating with multiple stakeholders to develop a counselling intervention acceptable to facility- and community-based role-players. These processes may be useful for intervention adaptations in other LMIC settings.

## Limitations

The search for manuals was based on a structured review of the literature but was not systematic. Therefore, we may have missed examples of perinatal task sharing counselling manuals from LMIC. The intervention content was developed specifically for low-resourced settings in South Africa and may require adaptations for use in other LMICs. Our study emphasises the importance of supervision and we found that even though we could recommend selection criteria for CHWs, this option was not possible for supervisors. We found that an aspect that requires further research attention involves suitable training and supervision for supervisors, within health system processes.

## Conclusions

A need exists to introduce a simple short-term, evidence-based, task-sharing perinatal psychological intervention to treat mild to moderate symptoms of perinatal depression and anxiety, as part of routine primary health care. The newly introduced National Community Health Worker Programme provides a suitable framework to introduce a brief 3-session psychological intervention, standardised training and supervision practices as part of routine community-based health system processes. Multi-sector stakeholder engagements were conducted to understand health system requirements and adapt counselling content to ensure relevance, acceptability and buy-in from health service providers and users alike. Further implementation and a formal evaluation will form part of a pilot trial in the Cape Town metro.

## Data Availability

The University of Cape Town (UCT) processed personal data under the General Data Protection Regulation (GDPR). Consent for the use of personal data was obtained from participants, as detailed above. The data controller for the ASSET project is King’s College in London. Questions, comments and requests about data can be sent to the King’s College London Data Protection Officer Mr Albert Chan info-compliance@kcl.ac.uk.
